# Data management, documentation and analysis systems in radiation oncology: a multi-institutional survey

**DOI:** 10.1186/s13014-015-0543-0

**Published:** 2015-11-16

**Authors:** Kerstin A. Kessel, Stephanie E. Combs

**Affiliations:** Department of Radiation Oncology, Technische Universität München (TUM), Ismaninger Straße 22, 81675 Munich, Germany; Institute of Innovative Radiotherapy (iRT), Helmholtz Zentrum München, Ingolstädter Landstraße 1, Neuherberg, Germany

**Keywords:** Data management, Documentation system, Data Analysis, Electronic data capture, Survey

## Abstract

**Introduction:**

Recently, information availability has become more elaborate and widespread, and treatment decisions are based on a multitude of factors. Gathering relevant data, also referred to as Big Data, is therefore critical for reaching the best patient care, and enhancing interdisciplinary and clinical research. Combining patient data from all involved systems is essential to prepare unstructured data for analyses. This demands special coordination in data management. Our study aims to characterize current developments in German-speaking hospital departments and practices. We successfully conducted the survey with the members of the Deutsche Gesellschaft für Radioonkologie (DEGRO).

**Methods:**

A questionnaire was developed consisting of 17 questions related to data management, documentation and clinical trial analyses, reflecting the clinical topics such as basic patient information, imaging, follow-up information as well as connection of documentation tools with radiooncological treatment planning machines.

**Results:**

A total of 44 institutions completed the online survey (University hospitals *n* = 17, hospitals *n* = 13, practices/institutes *n* = 14). University hospitals, community hospitals and private practices are equally equipped concerning IT infrastructure for clinical use. However, private practices have a low interest in research work. All respondents stated the biggest obstacles about introducing a documentation system into their unit lie in funding and support of the central IT departments. Only 27 % (12/44) of responsible persons are specialists for documentation and data management.

**Conclusion:**

Our study gives an understanding of the challenges and solutions we need to be looking at for medical data storage. In the future, inter-departmental cross-links will enable the radiation oncology community to generate large-scale analyses.

**Electronic supplementary material:**

The online version of this article (doi:10.1186/s13014-015-0543-0) contains supplementary material, which is available to authorized users.

## Introduction

In the age of intelligent information systems, data sharing in a medical environment remains a challenging objective [1]. Therefore, the aim of researchers lies in ensuring that system architecture, data protection, communication protocols and usable procedures facilitate the communication of data for any use, regardless of its point of origin. This communication refers to the use and reuse of data by other systems in the same department, healthcare networks (e.g. for telemedicine consultations and clinical referral), or collaborative research projects.

In radiation oncology, we have recognized for years now the increasing scope offered by medical and technical progress and the associated demands on IT solutions and employees. The last decades showed enormous advances, for instance introducing particle therapy into clinical routine [2], dynamic treatment techniques using 4D imaging, or the development of MR-guided radiotherapy [3].

Recently, information availability has become more elaborate and widespread, and treatment decisions are based on a multitude of factors including imaging, molecular or pathological markers, surgical results and patient’s preference. Clinical data in general doubles in less than five years; 80 % of this data are unstructured [4]. Imaging as high dimensional data allocates a major share of healthcare storage space. Radiation oncology is a highly image intensive medical specialty. Diagnostic and therapeutic data are acquired throughout the course of treatment and during follow-up. Hence, in this interdisciplinary setting not only a heterogeneous and voluminous amount of data has to be evaluated, it is also spread in different styles across various information systems. Involved systems are the Hospital-, Laboratory- and Oncology Information System (HIS, LIS, OIS), Picture Archiving and Communication System (PACS) and Record & Verify System. The need grows to access data quickly and provide it within interdisciplinary meetings, research or acute emergencies. Researchers need assistance in reusing the terabytes of invaluable information collected routinely into these separate information systems. These information hold hidden treasures [5].

Relying on various quantitative data information becomes a strong focus, as disease management steps into the era of modern personalized and individualized medicine [6], thus involving the active contribution of multiple medical specialties. Gathering relevant data, also referred to as Big Data, is therefore critical for reaching the best clinical performance in patient care, and enhancing interdisciplinary and clinical research - ultimately leading to optimizing treatment concepts, adjusting them, and developing new ones. Combining patient data from involved systems is essential to prepare unstructured data for analyses. This demands special coordination in data management end electronic data capture (EDC) [7, 8] while at the same time complying with data protection regulations. Clinical data management systems or databases are therefore on the rise starting from different initial situations, aiming for different goals and following different approaches to cope with Big Data [9–13].

The data tools and systems available at different radiation oncology centers are heterogeneous. To understand where the radiooncological community stands and what recommendations could possibly be given, the present conditions of data management, documentation and analysis systems are determined. For this reason, this study aims to characterize current developments in German-speaking hospital departments and practices. We successfully conducted the survey with the members of the Deutsche Gesellschaft für Radioonkologie (DEGRO).

## Methods

A questionnaire was developed by the department of Radiation Oncology, Technische Universität München (TUM), Klinikum rechts der Isar, Munich, based on the authors’ expert knowledge on establishing documentation systems in radiation oncology [14, 15]. The survey consisted of 17 questions related to data management, documentation and clinical trial analyses, reflecting the clinical topics such as basic patient information, imaging, follow-up information as well as connection of documentation tools with radiation oncology treatment planning machines (see Additional file 1 and 2) . Additionally, the research focus was analyzed, which is mostly connected to a university setting compared to a private practice. The questions were tailored for these two needs in a simple online-based setup. No weighting factors were assigned to adjust for center size, personnel and financial resources of the institutions. The survey content fell into the following categories: demographics or department information, existing hardware/software solutions, general scope and workflow of documentation and analyses, interfaces/communication standards, data maintenance responsibilities, big data and cloud solutions. We used a survey design with which all questions were visible on one side and visitors were able to see the whole scope of the survey.

As a first phase of the project, the survey was evaluated and cross-checked by ten experienced persons in the field of radiation oncology (physics, biology, medicine, informatics) to determine whether the questions asked are clear, understandable, and the answers provided are comprehensible. Only minor modifications were made to provide a user-friendly question format which lead to change of single selection to multiple selection questions. The authors distributed it as an online survey (Survio s.r.o., Czech Republic) via email to the members of DEGRO representing a substantial number of radiooncological (university) hospitals and practices in German-speaking countries. Participation was encouraged through repeated email reminders. The survey required approximately ten minutes to complete. The survey was carried out for four weeks.

## Results

After a period of four weeks in January and February 2015, a total of 44 German-speaking institutions (German: 42, Swiss: 2) completed the online survey (University hospitals *n* = 17, hospitals *n* = 13, practices/institutes *n* = 14). A return rate is not calculable as we sent the survey using the DEGRO email distributor. It is not possible to give an exact number of centers that were contacted. However, for the university hospitals we know that about 33 exist in Germany. Of all, 15 German university hospitals responded, which represents about 45 % (15/33) of the German university facilities.

Of all respondents 77 % (34/44) use a HIS for electronic documentation. Of those, 13 (38 %, 13/34) have an electronic health record (EHR) available within the HIS for data capture. An independent EHR system is available in 29 (66 %, 29/44) institutions used as an additional system (27 %, 12/44) or as the only system for patient documentation (39 %, 17/44). In two cases (5 %, 2/44) no EHR is established.

The five most named HIS solutions of the 34 departments having a HIS installed are SAP/Siemens IS-H*med (35 %, 13/34), Agfa ORBIS (26 %, 9/34), Nexus/HIS (9 %, 3/34), Siemens Medico (6 %, 2/34) and iSoft Clinical Centre (6 %, 2/34). As for the PACS systems, the five most common systems in use are Agfa IMPAX (20 %, 9/44), GE Centricity PACS (16 %, 7/44), Siemens Syngo PACS (11 %, 5/44), Cerner ProVision PACS (5 %, 2/44) and Visus View PACS (5 %, 2/44). Mosaiq (52 %, 23/44) and Varian Aria (32 %, 14/44) as an OIS are mostly installed in radiooncological departments and practices.

All respondents participate in at least one of the following documentation projects: multi-center clinical trials (77 %, 34/44), central databases, such as ESTRO, DEGRO, EORTC, DKTK etc. (48 %, 21/44), or cancer registries/tumor documentation (91 %, 40/44).

We asked all institutions if they use self-reported outcome in their clinical routine. Firstly, we asked about patient self-reported outcome using general or standardized questionnaires such as EORTC QLQ-C30. Most use paper-based versions (59 %, 26/44); 11 (25 %, 11/44) use digital forms on a PC or web-based solution; 12 (27 %, 12/44) departments have no patient self-reported outcome implemented.

Secondly, in case of physician reported outcome, 64 % (39 % (17/44) hospitals, 25 % (11/44) practices) stated that they did not establish it, whereas 11 (25 %, 11/44) use paper-based, and 5 (11 %, 5/44) digital documentation in their department.

The tools used for documentation in terms of prospective/retrospective trials and evaluations are: paper (68 %, 30/44), Excel (77 %, 34/44), Access (25 %, 11/44) and special documentation/analysis systems such as eCRFs for certain trials (52 %, 23/44). In terms of documentation/analysis systems, the distribution of the 23 running systems for the different institution types is: 65 % (11/17) university hospitals, 38 % (5/13) hospitals and 50 % (7/14) practices.

**Fig. 1:**
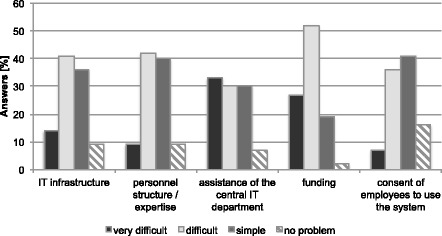
Response profile to the question “How would you assess the following obstacles regarding the implementation of a system for data management, documentation and analyses in your department?”

All respondents rated the obstacles about introducing a documentation system into their unit, see Fig. 1. Funding is the most difficult issue stated as very difficult by 27 % (12/44) and as difficult by 52 % (23/44). IT infrastructure, personal expertise and the consent of employees to use the system were similarly rated as difficult to easy. The support of the central IT department is the issue most reported as very difficult (32 %, 14/44), however, the same amount rated it as difficult (30 %, 13/44) or easy (30 %, 13/44).

Table 1 gives an overview on the availability of systems for documentation and electronic data capture in different application scenarios. The used or planned interfaces and communication standards can be seen in table 2, whereas the data transferred via these protocols are listed in table 3.

**Table 1:** Systems for documentation/EDC in use or planned for the following application scenarios

	System already implemented	System planned	Not planned / Not necessary
for prospective clinical trials / scientific evaluations, n (%)	15 (34 %)	8 (18 %)	21 (48 %)
for retrospective clinical trials / scientific evaluations, n (%)	15 (34 %)	9 (20 %)	20 (45 %)
for research documentation, n (%)	10 (23 %)	11 (25 %)	23 (52 %)
for clinical routine documentation, n (%)	30 (68 %)	8 (18 %)	7 (16 %)
for data backup, n (%)	35 (80 %)	3 (7 %)	6 (14 %)
for quality assurance, n (%)	28 (64 %)	8 (18 %)	8 (18 %)

**Table 2:** Interfaces / communication standards used or planned

	Already implemented	Planned	Not planned
HL7 (ADT, ORU, DFT,…), n (%)	19 (45 %)	7 (16 %)	17 (39 %)
DICOM (PACS…), n (%)	39 (89 %)	2 (5 %)	3 (7 %)
HTML, n (%)	20 (45 %)	2 (5 %)	22 (50 %)
HTTPS, n (%)	13 (30 %)	5 (11 %)	26 (59 %)
webservices, n (%)	15 (34 %)	4 (9 %)	25 (57 %)
FTP, n (%)	7 (16 %)	2 (5 %)	35 (80 %)
IHE conformity, n (%)	3 (7 %)	3 (7 %)	38 (86 %)

**Table 3:** Data transferred via interfaces / communication standards

	Already implemented	Planned	Not planned
imaging data (diagnostic and therapeutic), n (%)	39 (89 %)	2 (5 %)	3 (7 %)
radiation data (RT Plan, RT Dose, RT StructureSet), n (%)	32 (73 %)	4 (9 %)	8 (18 %)
surgical findings, n (%)	23 (52 %)	5 (11 %)	16 (36 %)
lab findings, n (%)	29 (66 %)	4 (9 %)	11 (25 %)
pathology findings, n (%)	25 (57 %)	5 (11 %)	14 (32 %)
follow-up information, n (%)	21 (48 %)	12 (27 %)	11 (25 %)
clinical trial data, n (%)	17 (39 %)	12 (27 %)	15 (34 %)

About half of all respondents are not installing systems for prospective (48 %, 21/44) and retrospective clinical trials (45 %, 20/44) as well as research documentation (52 %, 23/44) (table 1). Most negative answers are solely from practices: 18 % (8/44), 16 % (7/44), 23 % (10/44).

**Fig. 2:**
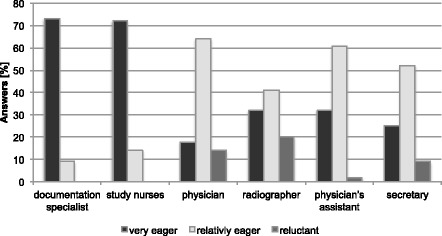
Response profile to the question “How do you rate the willingness of the following groups of people to use a documentation / EDC system?”

All respondents rated the willingness of different user groups to use a documentation/EDC system, see Fig. 2. Clearly, documentation specialist and study nurses are seen as the main responsible persons and rated with a high willingness for documentation >70 %. Most reluctant towards documentation are radiographers (20 %, 9/44). Physicians (64 %, 28/44), physician’s assistants (61 %, 27/44)) and secretaries (52 %, 23/44) build up the midfield being relatively eager to document.

The responsible person(s) managing system(s) for EDC/documentation/analyses are mostly physicists (50 %, 22/44) doing the IT job in the department/practice, followed by IT specialists (27 %, 12/44), physicians (7 %, 3/44) and documentation specialists (5 %, 2/44). Five respondents made no entry for this question (11 %, 5/44). Of all, 27 % (12/44) are primarily responsible for the IT support in their department. These are the same 27 %, who are employed as IT specialists; 68 % (30/44) are otherwise employed as physicians and physicists with several other tasks and manage the documentation system(s) as their secondary task -; in two cases (5 %, 2/44) responsibilities are unknown.

The last questions concern data handling in the future in terms of big data and cloud solutions. The majority of 56 % (25/44) has already heard of the term “big data” in the context of health care. Of those, 13 (30 %, 13/44) have taken no measures so far, 9 (20 %, 9/44) have only started theoretical discussions, and 3 (7 %, 3/44) work on implementations by building interface structures and standardized data storage or have approaches for solely scientific use. Cloud solutions are only used in 4 of all 44 institutions. One practice is using dropbox for international therapy requests and image sharing, the remaining three use cloud solutions only for scientific use.

## Discussion

This article reports on the results of a scientific online survey performed to inquire the current status on EDC and management, documentation and analysis systems in German-speaking radiooncological hospital departments and practices. Given our resources email was the most efficient, inexpensive and timely uncomplicated way of contacting survey participants. Online surveys facilitate fast and free collection and management of data.

Prospective and retrospective clinical trials as well as research documentation have a strong scientific background. Private practices have a low interest in research work but mostly performance-oriented. Consequently, they form the largest group not investing in systems for prospective and retrospective clinical trials as well as research documentation (see table 1). However, they could provide an enormous data pool for large-scale treatment analyses. Therefore, the radiation oncology community should invest into collaborative research-networks.

University hospitals, community hospitals and private practice groups are equally equipped concerning IT infrastructure for clinical use. Now is the time to encourage and establish the development of data management, documentation and analysis systems, however, the biggest problems lie in initial funding and recurring cost for support and upgrades as well as for employees necessary to maintain such a system. Further, one recipient stated that concerns about documentation reliability still exists compared to old paper-based documentation. Another stated that the expertise and clear responsibilities for data, data sharing are unclear in its department. As previously published [14], system maintenance is an important profession in documentation intensive fields such as radiation oncology. It is important to train specialists for management and coordination of clinical data. Our results show that all institutions lack these experts. Only 27 % (12/44) of responsible persons are in some way specialists for documentation and data management. With the increasing data volumes, these positions are getting more and more important, and should be included routinely into hospital environments. In German structures for Oncology (Comprehensive Cancer Center (CCC)), it is already established that clinical data is collected and specific documentation assistants regularly manage patient data prospectively.

Often patients go to their local oncologist for check ups after radiation therapy, due to a greater distance to the radiation center. Follow-up tracking is highly important in radiation oncology to study the long-term therapeutic effect, or to detect recurrences early and have an excellent chance of cure. 39 % (17/44) of hospitals do not use any kind of physician reported outcome. Naturally, it is necessary to be able to follow the course of treatment at all times in order to improve and provide optimal patient care [16] and analyze long-term treatment effects. Retrospective analyzes are, among other things, very time consuming because of the additional data acquisition afterwards [15]. A continuous documentation and data pooling in cooperation with all involved parties is therefore requested.

Analysis data no longer fits in a simple Excel sheet [17]. In scientific journals and at relevant conferences “Big Data” is currently one of the hottest topics. However, it is hard to keep up with new technologies in the clinical routine. As seen in the results, 7 % (3/44) only started on implementing solutions to cope with Big Data. Data confidentiality and security are of great relevance and solutions are currently not possible.

Apart from the valuable aim to gain knowledge from Big Data, a factor, which is not to be neglected, is the willingness of sharing all the data regardless of any data protection issues. We experienced before [14] the reluctance in sharing data within multicenter studies or even within an institution between various departments. It is often the individual, who in the way being possessive of its supposedly own data. It is up to the head of departments, to solve this problem and to impose and exemplify data sharing.

As medical data explodes in volume, data protection challenge gets greater and greater. In terms of cloud computing, one recipient stated exactly that the IT department is not allowing any technical implementation due to data privacy and security. Additional, substantial and continuous resource investments are needed to proceed in this area. However, we need to introduce sophisticated, new technologies into the routine to improve patient safety and treatment outcomes. One practice is using dropbox for international therapy requests, which offers no level of patient data privacy protection. Obviously, data is often not managed by experts in many facilities. Again, these experts need to be employed to define data protection, storage and exchange strategies and establish data interoperability for intelligent data management. This allows data to be cross-correlated with increasingly advanced laboratory, clinical and genomic/proteomic information. With this we can start in tomorrow’s practice of medicine.

## Conclusion

Our survey shows the current status on EDC and management, documentation and analysis systems in German-speaking radiooncological hospital departments and practices. The data show clearly that in both university as well as community based hospitals and private practice radiation oncology structures are equipped with modern data storage software, in all scenarios at least for clinical use. It gives us an understanding of the sorts of challenges and solutions that we need to be looking at for medical data storage. For the university centers, a subgroup is specialized in research oriented database structures. In the future, inter-departmental cross-links will enable the radiation oncology community to generate large-scale analyses for upcoming research topics. In this context, education of documentation specialists is essential to guarantee that all data is protected and reliably available within the systems.

## Additional files

Additional file 1:
**Questionnaire in German.** (PDF 116 kb) Additional file 2:
**Questionnaire in English.** (PDF 116 kb)
