# Supplementary material for: Data management, documentation and analysis systems in radiation oncology: a multi-institutional survey
Source: Radiat Oncol. 2015 Nov 16;10:230. doi: 10.1186/s13014-015-0543-0 (PMC4647666; doi:10.1186/s13014-015-0543-0)
Supplement: Additional file 1: — Questionnaire in German. (PDF 116 kb) [file 13014_2015_543_MOESM1_ESM.pdf]

Dokumentations- / Studien- / Auswertesysteme in der RadioOnkologie

Liebe Kolleginnen und Kollegen der Radioonkologie,

zum Thema **Studien und Auswertungen in der Radioonkologie** und den damit verbundenen **Dokumentations- bzw. EDC Systemen (electronic data capture = elektronische Datenerfassung)** haben wir folgende Umfrage erstellt. Wir würden uns freuen wenn Sie sich zum Ausfüllen 10 Minuten Zeit nehmen.

Die meisten Fragen enthalten Pflichtfelder. Sie müssen „-" eingeben, wenn Sie keine Angaben machen können/wollen. Es ist ggf. hilfreich, wenn ein Mitarbeiter der Physik/IT beim Ausfüllen behilflich ist, da Fragen zu Schnittstellen Spezialkenntnisse im IT-Bereich voraussetzen.

Über die Ergebnisse werden wie Sie gerne informieren.

Univ.-Prof. Dr. med. Stephanie E. Combs   Dr. sc. hum. Kerstin A. Kessel

1

Nennen Sie Ihre Abteilung und Kontaktinformationen:

Abteilung

Name Kontaktperson

E-Mail Kontaktperson

2

Elektronische Patientenakte (EPA): Was trifft zu? (Mehrfachantworten möglich)

- ☐ Unsere Abteilung nutzt das zentrale Klinikinformationssystem (KIS) zur elektronischen Dokumentation.
- ☐ Unsere Abteilung führt innhalb des KIS eine elektronische Patientenakte (EPA).
- ☐ Unsere Abteilung nutzt eine elektronische Patientenakte (EPA) unabhängig vom KIS.
- ☐ Eine elektronische Patientenakte (EPA) ist bei uns nicht etabliert.

3

Welche Hard-/Software-Lösungen welcher Firmen nutzt Ihre Abteilung für ...

	bitte machen Sie eine Angabe für jedes Feld
KIS (Krankenhausinformationssystem)	
RIS (Radiologieinformationssystem)	
PACS (Picture Archiving and Communication System)	
LIS (Laborinformationssystem)	
OIS (Onkologieinformationssystem z.B. Mosaiq, Lantis...)	

4

An welchen zentralen Dokumentationsprojekten beteiligt sich Ihre Abteilung?

- ☐

- [ ] Dokumentation in zentrale Datenbanken wie z.B. der ESTRO, EORTC...
- [ ] multizentrischen Studien
- [ ] Krebsregister / Tumordokumentation
- [ ] andere

5

Nutzen Sie "self-reported outcome" in Ihrer Abteilung?

	ja, als Papierformular	ja, als digitales Formulare (z.B. am PC)	ja, als webbasiertes Formular	aktuell nicht
"patient self-reported outcome" = allg. Fragebögen / standardisierte QLQ Fragebögen (z.B. EORTC QLQ-C30)	☐	☐	☐	☐
"physician reported outcome" von Zuweisern	☐	☐	☐	☐

6

Welche Dokumentationsvarianten nutzen Sie in Ihrer Abteilung für prospektive / retrospektive Studien / Auswertungen?

- [ ] Papier
- [ ] Excel
- [ ] Access
- [ ] spezielles Dokumentation- / Auswertessystem
- [ ] andere

Wie würden Sie die folgende Hürden einschätzen bzgl. der Einführung eins Dokumentationssystems für Studien und Auswertungen in Ihrer klinischen Abteilung?

	sehr schwierig	schwierig	einfach	kein Problem
IT Infrastruktur	○	○	○	○
Personelle Struktur / Expertise	○	○	○	○
Unterstützung des zentralen Rechenzentrums	○	○	○	○
Finanzierung	○	○	○	○
Zustimmung von Mitarbeitern (u.a. Ärzten, study nurses etc.) in das System zu dokumentieren	○	○	○	○

Werden in Ihrer Abteilung für folgende Anwendungsszenarien Dokumentations- oder EDC (electronic data capture = elektronische Datenerfassung) Systeme eingesetzt oder planen Sie ein System in der Zukunft einzusetzen?

	System ist bereits vorhanden	System ist geplant	nicht geplant/ notwendig
für prospektive wissenschaftliche Studien / Auswertungen	☐	☐	☐
für retrospektive	☐	☐	☐

wissenschaftliche Auswertungen / Evaluationen			
zur Forschungsdokumentation	☐	☐	☐
in der klinischen Routine	☐	☐	☐
zur Datensicherung	☐	☐	☐
zum Qualitätsmanagement	☐	☐	☐

9

Gibt es andere Systeme, für jegliche Dokumentation, die aktuell bzw. zukünftig eingesetzt werden?

10

Wenn Sie an andere Systeme im Kliniknetz denken, welche Schnittstellen / Kommunikationswege zum Dokumentationssystem werden genutzt oder sind geplant?

	bereits umgesetzt	in Planung	nicht in Planung
HL7 (ADT, ORU, DFT,...)	○	○	○
DICOM (PACS...)	○	○	○
HTML	○	○	○
HTTPS	○	○	○
Webservices	○	○	○
FTP	○	○	○
IHE Konformität	○	○	○

11

Welche Informationen werden über die Schnittstellen / Kommunikationswege übertragen?

	bereits umgesetzt	in Planung	nicht in Planung
Bilddaten (diagnostisch u. therapeutisch)	○	○	○
Bestrahlungsdaten (RT Plan, RT Dose, RT StructureSet)	○	○	○
OP Berichte	○	○	○
Laborbefunde	○	○	○
Pathologiebefunde	○	○	○
Nachsorgedaten	○	○	○
Studienrelevante Daten	○	○	○

12

Bezüglich der letzten beiden Fragen: Welche andere Schnittstellen / Kommunikationswege werden in Ihrer Abteilung noch genutzt oder sind geplant? Welche anderen Daten werden auch übertragen?

13

Wie beurteilen Sie die Bereitschaft folgender Personengruppen ein Dokumentationssystem zu nutzen?

	sehr willig	teilweise	unwillig	dokumentieren

		willig		nicht
spezielle Dokumentare	○	○	○	○
Study nurses	○	○	○	○
Ärzte	○	○	○	○
MTRA	○	○	○	○
Arzthelfer/in	○	○	○	○
Sekretär/in	○	○	○	○
andere Mitarbeiter	○	○	○	○

14

Die zuständige Person / zuständigen Personen, welche die Systeme betreuen, ...

	bitte machen Sie eine Angabe für jedes Feld
haben welche Ausbildung bzw. welchen Abschluss?	
bringen welche besondere Expertise mit?	
haben die Systembetreuung als Nebenaufgabe oder sind alleinig dafür eingestellt?	

15

Haben Sie schon von dem Begriff "Big Data" im Kontext von Gesundheitswesen gehört?

○ ja

○ nein

16

Big Data in der RadioOnkologie:

bitte machen Sie eine Angabe für jedes Feld

In welchem Umfang hat sich Ihre Abteilung damit schon beschäftig?

Welche Maßnahmen planen Sie in der Zukunft?

17

Nutzen Sie bereits Cloud-Lösungen in Ihrer Abteilung?

- ja
- nein
- in Planung

18

Cloud Lösungen in der RadioOnkologie:

bitte machen Sie eine Angabe für jedes Feld

Welche Lösungen nutzen Sie?

Für welchen Szenarien?

19

Haben Sie noch Kommentare zu unserer Umfrage?

ABSENDEN

Kostenlos **Umfrage erstellen** (http://www.survio.com/de/?source=survey_footer&medium=link&term=survey_link) ✓
Powered by Survio (http://www.survio.com/de/merkmale-funktionen?source=survey_footer&medium=link&term=brand)
