# Supplementary material for: Data management, documentation and analysis systems in radiation oncology: a multi-institutional survey
Source: Radiat Oncol. 2015 Nov 16;10:230. doi: 10.1186/s13014-015-0543-0 (PMC4647666; doi:10.1186/s13014-015-0543-0)
Supplement: Additional file 2: — Questionnaire in English. (PDF 116 kb) [file 13014_2015_543_MOESM2_ESM.pdf]

Data management, documentation and analysis systems in radiation oncology

Dear colleagues in radiation oncology,

on the subject of **data management**, **documentation** and **trial analyses in radiation oncology** and thus **EDC systems** (electronic data capture), we have created the following survey. Please participate and take 10 minutes to complete the survey.

Most of the questions are mandatory. You need to enter "-" if you don't want to / can't make any comments. It might be helpful if an employee of the Physics / IT department is present as some questions about interfaces require special knowledge in the IT field.

We are pleased to inform you about the results.

Univ.-Prof. Dr. med. Stephanie E. Combs   Dr. sc. hum. Kerstin A. Kessel

1

Name your department and contact information:

your department (name and country)	
your name	
your email address	

2

Electronic Health Record (EHR): What is correct? (Multiple answers possible)

- [ ] Our department uses the central hospital information system (HIS) for electronic documentation.
- [ ] Our department has an electronic health record (EHR) within the HIS.
- [ ] Our department uses an EHR independently from the HIS.
- [ ] An EHR is not established in our department.

3

What kind of hardware / software solutions (company name) does your department use for ...

	please fill out each field
HIS (hospital information system)	
RIS (radiology information system)	
PACS (picture archiving and communication system)	
LIS (laboratory information system)	
OIS (oncology information system e.g. Mosaiq, Lantis...)	

4

In which central documentation projects does your department participate in?

- [ ] documentation in central databases, such as ESTRO, EORTC...

- [ ] multi-center clinical trials
- [ ] cancer registries / tumor documentation
- [ ] other

Type your answer

100 characters remaining

5

Do you use "self-reported outcome" in your department?

	yes, as paperbased forms	yes, as digital forms (e.g. on a PC)	yes, as webbased forms	not currently
"patient self-reported outcome" = gen. questionnaires / standardized questionnaires about quality of life (e.g. EORTC QLQ-C30)	☐	☐	☐	☐
"physician reported outcome" of referring physicians	☐	☐	☐	☐

6

What tools for documentation do you use in your department for prospective / retrospective trials / evaluations?

- [ ] paper
- [ ] Excel
- [ ] Access
- [ ] special documentation / analysis system
- [ ] other

Type your answer

100 characters remaining

7

How would you assess the following obstacles regarding the implementation of a system for data management, documentation and analyses in your department?

	very difficult	difficult	simple	no problem
IT infrastructure	○	○	○	○
personnel structure / expertise	○	○	○	○
assistance of the central IT department	○	○	○	○
funding	○	○	○	○
consent of employees (including physicians, study nurses, etc.) to use the system	○	○	○	○

8

Are systems for documentation / EDC in use or planned for the following application scenarios?

	system already implemented	system in planning stage	not planned / not necessary
for prospective clinical trials / scientific evaluations	☐	☐	☐
for retrospective clinical trials / scientific evaluations	☐	☐	☐
for research documentation	☐	☐	☐

for clinical routine documentation	☐	☐	☐
for data backup	☐	☐	☐
for quality assurance	☐	☐	☐

9

Are other systems for any type of documentation used or planned in the future?

Type a sentence

250 characters remaining

10

If you think of other systems in your clinical environment, which interfaces / communication standards are used or planned for documentation?

	already implemented	in planning stage	not planned
HL7 (ADT, ORU, DFT,...)	○	○	○
DICOM (PACS...)	○	○	○
HTML	○	○	○
HTTPS	○	○	○
Webservices	○	○	○
FTP	○	○	○
IHE conformity	○	○	○

11

What information is transferred via the interfaces / communication standards?

	already implemented	in planning stage	not planned
imaging data (diagnostic and therapeutic)	○	○	○
radiation data (RT Plan, RT Dose, RT StructureSet)	○	○	○
surgical findings	○	○	○
lab findings	○	○	○
pathology findings	○	○	○
follow-up information	○	○	○
clinical trial data	○	○	○

12

With respect to the last two questions: What other interfaces / communication standards are used / planned in your department? What other data is also transferred?

Type a sentence

250 characters remaining

13

How do you rate the willingness of the following groups of people to use a documentation / EDC system?

	very eager	relatively	reluctant	not

		eager		documenting
documentation specialist	◯	◯	◯	◯
study nurses	◯	◯	◯	◯
physician	◯	◯	◯	◯
radiographer	◯	◯	◯	◯
physician's assistant	◯	◯	◯	◯
secretary	◯	◯	◯	◯
other employees	◯	◯	◯	◯

14

The responsible person(s) managing system(s) for EDC / documentation / analyses in your department ...

	please fill out each field
completed what kind of training or has/have what degree?	
has/have special expertise in?	
manges/manage the system(s) as a primary or secondary task?	

15

Have you heard of the term "Big Data" in the context of health care?

◯ yes

◯ no

16

Big Data in radiation oncology:

please fill out each field

To which extent has your department dealt which the subject of Big Data?

What measures do you plan in the future?

17

Is your department already using cloud solutions?

- yes
- no
- in the planning stage

Type your answer

***100** characters remaining*

18

Cloud solutions in radiation oncology:

please fill out each field

What solutions do you use?

For which scenarios?

19

Do you have any comments about our survey?

Type a paragraph

1500 characters remaining

SUBMIT SURVEY

It is easy to **create a survey (http://www.survio.com/en/?source=survey_footer&medium=link&term=survey_link)** free
✓ Powered by Survio (http://www.survio.com/en/features?source=survey_footer&medium=link&term=brand)
